# Brain Age Prediction/Classification through Recurrent Deep Learning with Electroencephalogram Recordings of Seizure Subjects

**DOI:** 10.3390/s22218112

**Published:** 2022-10-23

**Authors:** Kameron Jusseaume, Iren Valova

**Affiliations:** Computer and Information Science Department, University of Massachusetts Dartmouth, Dartmouth, MA 02747, USA

**Keywords:** brain age classification, regression analysis prediction, long short-term memory, neural networks

## Abstract

With modern population growth and an increase in the average lifespan, more patients are becoming afflicted with neurodegenerative diseases such as dementia and Alzheimer’s. Patients with a history of epilepsy, drug abuse, and mental health disorders such as depression have a larger risk of developing Alzheimer’s and other neurodegenerative diseases later in life. Utilizing recordings of patients’ brain waves obtained from the Temple University abnormal electroencephalogram (EEG) corpus, deep leaning long short-term memory neural networks are utilized to classify and predict patients’ brain ages. The proposed deep learning neural network model structure and brain wave-processing methodology leads to an accuracy of 90% in patients’ brain age classification across six age groups, with a mean absolute error value of 7 years for the brain age regression analysis. The achieved results demonstrate that the use of raw patient-sourced brain wave information leads to higher performance metrics than methods utilizing other brain wave-preprocessing methods and outperforms other deep learning models such as convolutional neural networks.

## 1. Introduction

Electroencephalograms (EEG) are primarily used to monitor the brain activity of subjects who have suffered from brain-related disorders, ranging from epilepsy to stroke-induced brain lesions. An EEG can be used to diagnose other disorders affecting brain activity such as Alzheimer’s and narcolepsy. These issues appear more frequently as patients age. With the growth of the population and an increase in the average lifespan of human beings, there is a rising number of individuals with non-fatal disabling disorders. The occurrence of neurodegenerative diseases such as dementia is becoming more frequent. As people age, their ability to multitask decreases, coupled with lower situational-processing speeds. As the length of the natural lifespan increases, so does the risk of fatality via neurodegenerative disease.

Hence, the research has focused on studying patients’ brain ages using EEG signals. EEG recording devices can measure brain waves in different regions using electrodes attached to the scalp. EEG recording in general is the least expensive and least complex recording mechanism when compared to electrocorticography (ECoG), which places the nodes on an exposed surface of the brain, or functional magnetic resonance imaging (fMRI) devices. The EEG-recording process typically involves the patient reclining in a chair or bed while a select number of electrodes are placed on the scalp. Patients are asked to keep their eyes closed and remain still as the recording process proceeds, as any blinking or body movements could cause inaccuracies in a reading. The conductor of the EEG recording may ask patients to perform deep breaths or react to a flash a bright light after the initial recording in order to produce brain activity that is not present during rest. The EEG-recording times are dependent on the severity of the presented symptoms and may take between 45 min to 2 h. Neurologists are tasked with monitoring the patient’s brain waves during the test for abnormalities or critical events such as epileptic seizures or interictal spikes. However, due to the number of patients undergoing testing, neurologists often monitor many EEGs at the same time. Thus, the use of machine learning architectures for evaluating brain waves can assist in reducing the amount of work placed on neurologists, allowing for a much faster detection of seizure symptoms.

Brain waves are rhythmic or repetitive patterns within the central nervous system [1]. Brain waves, or neural oscillations, can serve various physiological functions and correlate with differences in behavioral states and can be modulated in space and time, as they fluctuate between up and down states. Up states are associated with firing bursts of action potentials, or times of attention, whereas down states are associated with resting activity in the brain. There are differences in the frequencies of brain waves, known as frequency bands. Researchers, using power spectrum analysis, have found that neuronal oscillations fluctuate between specific frequency bands, ranging from under 0.01 Hz to over 1000 Hz [2].

Various regions of the brain do not often emit the same brain wave frequency simultaneously and an EEG signal consists of many waves with different characteristics. These different characteristics and large numbers of data received from just one EEG recording can make interpretation difficult, as brain wave patterns are unique for every individual [3]. In patients with a history of seizures or epilepsy, the brain operates in what is known as interictal and ictal states. The interictal state is a seizure-free state, or the state in which the patient is not currently experiencing an epileptic attack, while the ictal state entails the state in which a patient is currently in the middle of a seizure. With regard to seizure patients, their brain waves can be even more diverse than others, as large recording spikes can vary in intervals depending on the severity of their symptoms or the test that was performed during recording time [4].

The changes in cerebral activity are dependent on age and can be used to study the chronological age of a patient and how it differs from their biological age. By utilizing recorded EEG signals and extracting these frequency bands, the difference between a patient’s chronological age and their biological brain age based on the brain signal power can be detected. The raw EEG signals that have not had these frequency bands extracted, as well as the use of specific frequency bands, are explored in this work, and their impacts on the results are noted in further sections.

This research focuses on raw EEG signals and extracted brainwave frequency bands to evaluate the effectiveness of deep learning architectures for both age classification and age regression analysis. The utilized deep learning models include long short-term memory (LSTM), gated recurrent units (GRU), and bidirectional LSTM (BLSTM) and bidirectional GRU (BGRU) architectures, which are compared with respect to both their results and efficiency. While all the models have been constructed and tested, the GRU-based variety achieved results very similar to LSTM and BLSTM, which prompted their exclusion in the relevant sections.

The use of deep learning models on data from different brain recording methods has been a recent point of interest for researchers. Many studies, such as Franke et al.’s [5], have utilized convolutional neural networks (CNNs) and T1-weighted MRI structural images to establish a framework for estimating the age of healthy individuals. Qin et al. [6] reported a mean absolute error (MAE) of 4.6 years between the chronological age and predicted age utilizing fMRI data. Lam et al. [7] proposed a 2D slice-based recurrent neural network model with two components to classify brain age based on MRI scans. Their model consists of two components: a 2D CNN to encode the features of the MRI scan and a RNN to learn the relationship between the MRI scan information, yielding an MAE of 2.8 years [7].

The traditional machine learning approaches, including support vector machines (SVM) [8], linear discriminant analysis (LDA) [9], random forest [10], or transformer-based models [11,12], attempt to extract relevant features based on assumptions. Therefore, some important features may be excluded during feature extraction. Deep learning models attempt to learn features that may not be possible to extract using traditional methods. The work performed in [13,14,15] explored the use of LSTM architectures to detect emotional changes based on EEG and facial expressions, lapses in mental states due to fatigue known as “microsleeps”, and recognize brain fog in students while watching recorded online course videos. In this work, we focus on the age prediction and classification of subjects considering the importance of this subject parameter in the evaluation of brain health.

Other experiments using CNNs include those by Truong et al. [16], who utilized a dataset of more than 1000 juvenile participants to evaluate the performance of multiple different CNN architectures on both preprocessed and raw EEG data for sex classification and prediction. Their results showed that the same CNN architectures with minimally processed EEG data could have greater results than with fully preprocessed EEG features [16]. A recent study [17] delved into drowsiness prediction based on a single channel of EEG through a CNN.

Researchers who utilized the Temple University abnormal EEG corpus (TUAB) and performed work related to age classification and prediction include Gemein et al. [18] and Engemann et al. [19]. The former compared end-to-end decoding using deep neural networks versus feature-based decoding and reported a result wherein the decoding accuracies of various EEG-decoding methods lie within the range of 80–86%. Though the focus was primarily on the more traditional machine learning approaches, their results show that the proposed feature-based decoding framework can achieve accuracies on the same level as the state-of-the-art deep neural networks for age classification and prediction. The latter work, that of Engemann et al., evaluated machine learning approaches for patient age prediction. The ShallowFBCSPNet (Schirrmeister et al. [20]) was used as a benchmark in this work, achieving an MAE error value of 8 years between a patient’s actual age and brain age. We utilize the comparative analysis in [19] to determine how well RNN architectures perform compared to CNNs in EEG-based age prediction.

Finally, research works that utilize LSTM-based architectures for EEG evaluation must be mentioned, as in the work of Hasib et al. [21], who used a Hierarchical Long Short-Term Memory (H-LSTM) model to predict human decisions based on continuous EEG signals. Their model can outperform a normal LSTM model by 12.4% and a shallow SVM model by 17.4%. Kaushik et al. [22] proposed a Deep BLSTM-LSTM for both gender and age classification and prediction from EEG signals utilizing a dataset of 60 individuals (35 Male and 25 Female), with ages ranging from 6 to 55 years old. The dataset of 60 individuals was then divided into six different age groups split by ~3–4-year periods for ages 6–55. Upon using discrete wavelet transformation to extract the frequency bands, the conclusion that beta brainwaves lead to the best accuracy overall for both the gender classification and age prediction of the individuals was reached. Our work records comparable or better accuracies in both prediction and classification based on the raw EEG data. Many research publications attempt to leverage the use of discrete wavelet transform (DWT) or fast Fourier transform (FFT) to preprocess the EEG signals for their use in deep learning applications. This process can involve a great deal of trial and error without an agreed upon metric or range of values to utilize to transform the brain signal information. This can lead to varying degrees of difference among research works that utilize DWT or FFT brain signal-processing techniques and those that do not. In the proposed framework, we focus purely on the power of the deep learning architectures to evaluate EEG signals with minor preprocessing performed, i.e., the raw patient EEG data are utilized.

The remainder of the paper presents an overview of the dataset obtained from the Temple University EEG Corpus and the preprocessing methods are also detailed along with the methods undertaken to form the neural network models. The experimental setup for each of the machine learning models, along with the results of those models’ performances with respect to the formed datasets, are noted in Section 3. The conclusions drawn regarding the utility of recurrent neural networks in the use of EEG data from this research and planned future improvements are noted.

## 2. Data

This section details the raw EEG data as well as any preprocessing steps applied either for normalization or attainment of the beta frequency bands. The set of major frequency bands—delta, theta, alpha, beta, and gamma—is noted by their frequency range. Though the exact ranges of these frequency bands differ between sources, delta brain waves have a frequency of ~(0.5–4) Hz and are most prominent during sleep or dreaming. The theta brain waves have frequencies of ~(4–8) Hz and are prominent in states of deep relaxation or drowsiness. Alpha brain waves have a frequency of ~(8–12) Hz and are prominent in passive attention modes or relaxation. Beta brain waves have a frequency of ~(12–35) Hz and are observed in a state of anxiety, or activity. Finally, gamma brain waves have a frequency between ~(35–50) Hz and are found in a state of high concentration.

### 2.1. The Temple University EEG Corpus

Temple University hosts a large database of recorded EEG signals for use in deep learning research in neuroscience. The current database hosts over 30,000 EEG recordings, with almost 17,000 EEG-recording sessions from approximately 11,000 unique subjects, spanning from 2002 to present day using multiple generations of EEG-recording equipment. The corpus contains EEG signal data stored in open-source European data format (EDF) files paired with text file EEG reports written by neurologists during each EEG recording session [23]. As for the demographics of the full dataset, it is composed of 51% female subjects with ages ranging from less than a year to over 90 years old, with an average age of 51. There are many datasets to choose from in the database, such as the abnormal EEG corpus (TUAB) with EEGs noted as either normal or abnormal based on a neurologist’s expertise. Another example is the artifact corpus (TUAR) used in classification projects that label EEG artifacts such as eye movement, chewing, or shivering. The Temple University EEG seizure corpus (TUSZ) contains annotated EEG recordings of points of seizures from EEG signals for use in deep learning research involving applications such as seizure detection.

**Figure 1:**
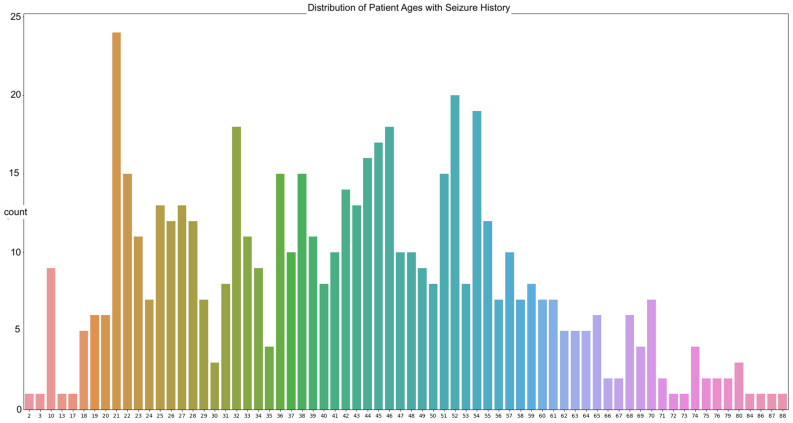
The distribution of patient ages from the utilized TUAB Corpus.

The data in our work are sourced from the Temple University abnormal dataset (TUAB). The goal is to predict the brain ages of those with histories of neurodegenerative diseases using raw EEG signals, with the focus on seizure patients due to the prevalence of those with seizure history in the dataset. A total of 564 patients in the TUAB dataset have a history of seizure or were tested for seizure activity. The TUAB dataset includes EEG signals recorded from patients of varying ages, from as young as 2 years of age to as old as 88. The average age of the patients in the recorded EEG signals utilized is 42 years of age (Figure 1).

**Figure 2:**
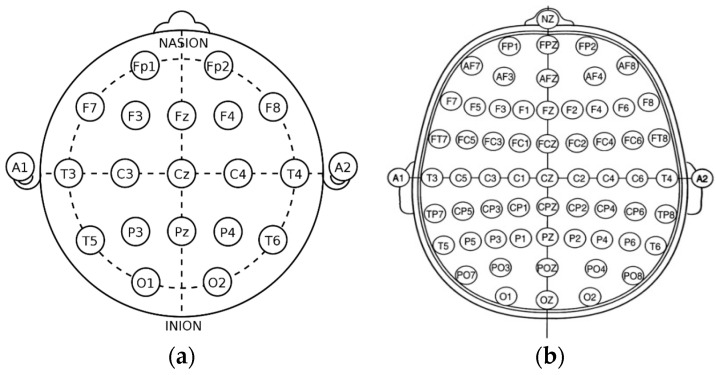
The basic view of the international system of EEG node placement: (**a**) 10/20 system; (**b**) 10/10 system.

The most frequently seen age in the dataset is 21 years of age with twenty-four occurrences. The signals were recorded using the international 10/20 system of EEG node placement (Figure 2). The length of the recordings spans a minimum of 30 min in the instance of a new patient to a maximum of 2–3 days based on severity of their condition. As per the Temple University data information document located in the database, 87% of the recorded EEG signals were recorded at 250 Hz, with the remaining data being sampled at 256 Hz, 400 Hz, or 512 Hz. This is important, as there is a need for a properly homogenized dataset. This abnormal dataset was split into both normal and abnormal EEG readings, such as those with a high Theta frequency or slower wave activity, thus requiring another scheduled recording. Therefore, the data that are utilized for these experimentations are those of the normal dataset, to avoid any potential error due to the recording mechanism or human interaction.

The abnormalities in the EEG recordings are stated in informational text files located together with each patient’s EEG recording EDF file. Each of these files contains information such as the date of the recording, general clinical information about the patient, whether they have a history of a particular disorder such as epilepsy or stroke, the patient’s age, any medications prescribed that the patient had taken prior to the examination, and a neurologist’s documentation regarding what was seen during the examination. Since the sessions cover a wide range of EEG recordings across the hospital [23], there is an abundance of channel-recording configurations as well as channel labels utilized to describe the EEG data.

### 2.2. The 10/20 System of EEG Node Placement and EEG Recording Overview

The 10/20 system of EEG node placement (Figure 2a) is based on the relationship between the location of an electrode and the underlying area of the cerebral cortex and is the most widely used arrangement of electrodes in EEG recording. Other variants of EEG node placement are the 10/10 (Figure 2b) and 10/5 system that provide less distance between the electrodes. The 10/20 system denotes that the distances between electrodes placed on the scalp are either 10% or 20% of the surface area of the skull, with the skull being divided into two sections across the middle, known as the nasion (front) and inion (back). The 10/20 system uses 4 anatomical landmarks for positioning of 21 electrodes: between the forehead and nose (nasion), the lowest point of the back of the skull (inion), and the preauricular areas near the front of the ears [23].

The data recorded in the EDF files within the EEG corpus consist of differential voltages in microvolts per millimeters.

The recorded voltages or signals obtained from the EEG process were saved as unipolar montages. Two general unipolar montages are used in the Temple University EEG Corpus: average reference (AR) and linked ears reference (LE). AR uses the average of a certain number of electrodes as a reference, while the LE uses a lead adapter to link the left and right ears of the patient to enable a stable reference point [23]. The unipolar montages are divided into 4 classifications, with the one being utilized in this paper being the 01_tcp_ar montage as labeled in the Temple University Dataset. This montage uses the AR-referencing method.

### 2.3. EEG Preprocessings

To ensure a homogenized dataset of similar brainwave distributions, patient brainwaves with the same diagnosis are used when training the deep learning models. Seizures are the most frequently occurring neurodegenerative condition at 87% in the full dataset, with the number of stroke patients placing second at 12%. The patient file name is utilized for categorization of the EEG signals after completion of preprocessing, and the patient age is extracted from the text files for classification or regression age prediction.

**Figure 3:**
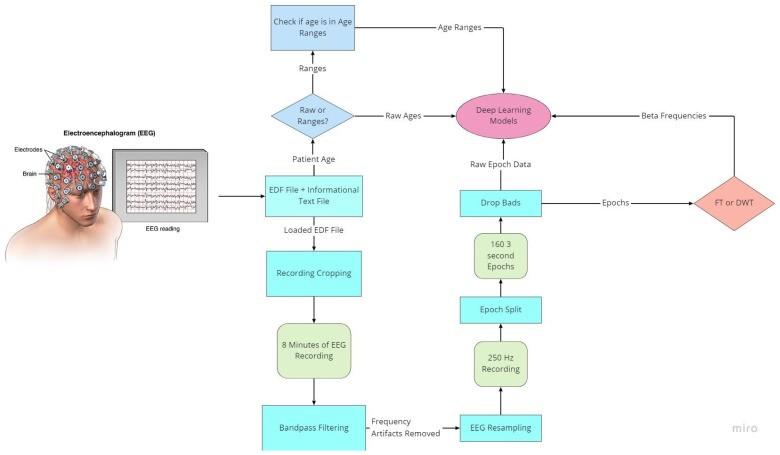
Flowchart of EEG preprocessing.

As with all deep learning works, preprocessing of data is the most important aspect to ensure data quality. EEG data are notably noisy and contain many recording artifacts, raising the importance of exploring the best preprocessing methods. EEG signals have specialized frequency bands that lie within specified frequency ranges. The preparation flowchart of the raw EEG signal is shown in Figure 3.

**Figure 4:**
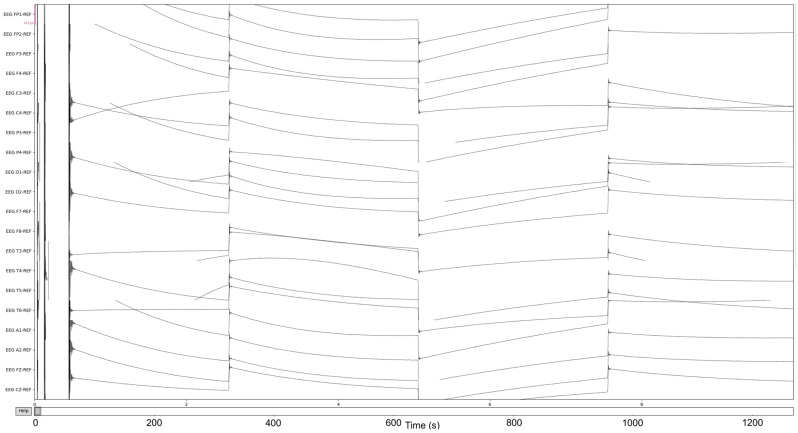
The pre-Reduction visualization of the EEG recordings.

To prepare for age prediction and classification, the two main goals of this research, EEG signals are sliced into sections of 8-minute recordings beginning after the first 60 s of recording. This is performed to avoid any potential human error from the start of recording and to ensure that large spikes of data in the initial period of recording would not hinder any preprocessing methods performed (Figure 4). The recorded EEG signals are then bandpass-filtered with a high pass-filter of 0.5 Hz to remove any low frequency artifacts, and a low pass filter of 120 Hz to remove any high-frequency artifacts or abnormalities. Next, the EEG recordings are resampled to 250 Hz to ensure a fully homogenized dataset, because, as stated, 87% of the data included in the dataset are recorded at 250 Hz, with some files being recorded at much higher frequencies.

The MNE-Python library is utilized for modification and visualization of EEG signals from the EDF files. Using the set of 23 commonly seen EEG signals from the 10/20 system of node placement, all other EEG node recordings are removed from the dataset. Specialized event-related potential (ERP) signals known as burst, photic stimulation (photic-ref), and interburst intervals (IBI) are also removed. These ERP signals evaluate the emotional intensity and response to external stimuli such as lights or any sounds used during the EEG-recording process. EKG-related recordings present in the dataset are also removed as they are used to monitor heart-related data, which is not the focus of this test. The EEG recordings are sliced into different fixed length epochs of recording times utilizing MNE-Python functionality. Using a frequency threshold, the epochs containing any large spikes of recording after the first 60 s are labeled as ‘bad’ epochs and removed from the dataset. The raw EEG recordings as well as the post-reduction visualizations of these datasets are visualized in step-by-step illustrations.

**Figure 5:**
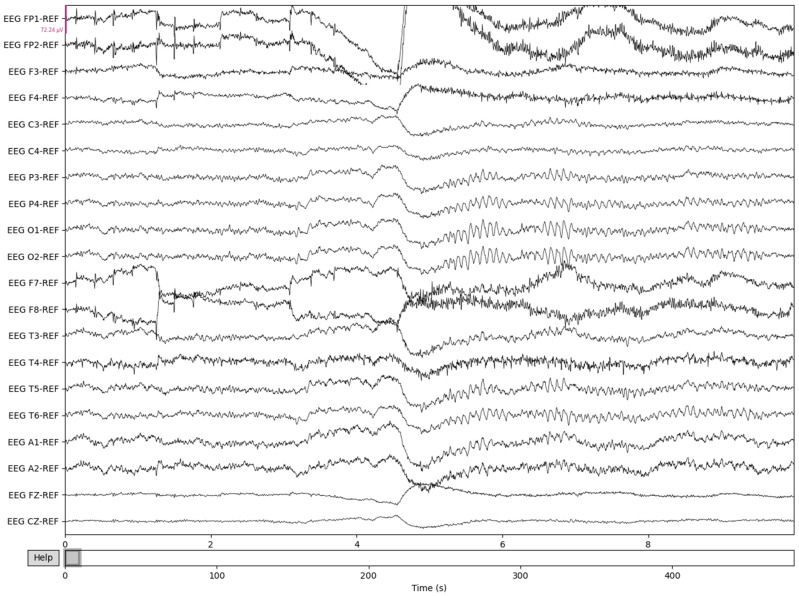
Post-cropping and resampling visualization of the EEG recordings.

The process is visualized through series of figures. The start illustrated in Figure 4 shows EEG recordings taken directly from the EDF files versus the post-cropping, filtering, and resampling from the dataset (Figure 5) of a 34-year-old man with histories of seizures. Each line represents one of the EEG recording nodes from the dataset and its signal over time measured in microvolts. Figure 4 displays the main reasoning for the cropping of the EEG recording time to 8-minute sections starting from the first 60 s, as the large, almost flatlined recordings of EEG signals had to be removed to ensure that they did not impede further processing. These large, flatlined recordings primarily appear in the first 60 s of the EEG recording process and after which the signals stabilized into what could be viewed as a normal EEG recording. Such recording blocks are a subject of debate with respect to their origin, as they could originate from human error when placing the EEG nodes on the subject’s scalp, the subject having their eyes open to stimuli in the initial stages of recording, or simply as a byproduct of the recording mechanism. The signal recordings resulting from the initial resampling and cropping can be seen in Figure 5, yielding what can be seen as a more normalized EEG recording.

**Figure 6:**
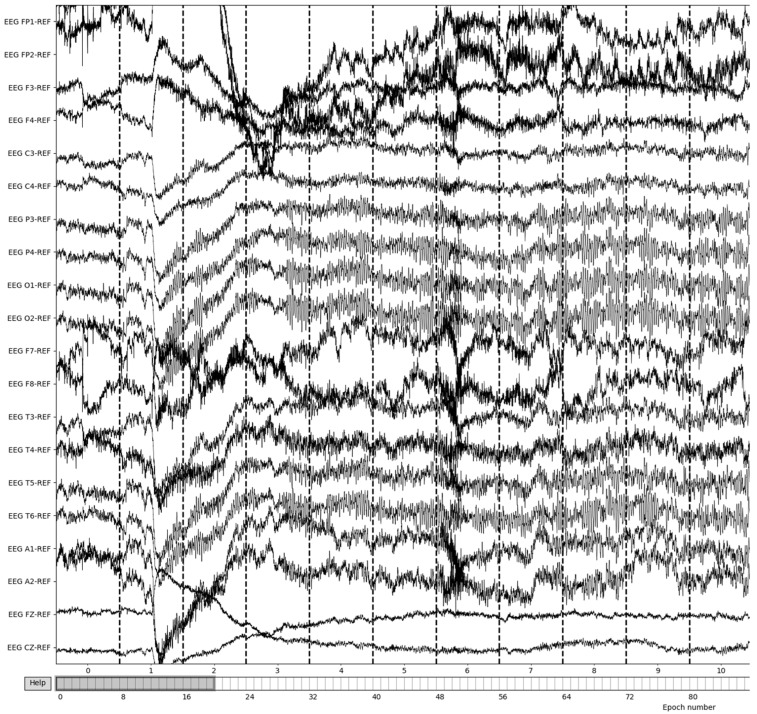
Creation of 3-second epochs of recordings.

The EEG data are then split into 3 s epochs to be evaluated for quality. With each epoch being 3 s of the 8-minute (480 s) recording, the process yields a total of 160 epochs of recording details. Figure 6 illustrates the results of 3-s epochs in recordings.

**Figure 7:**
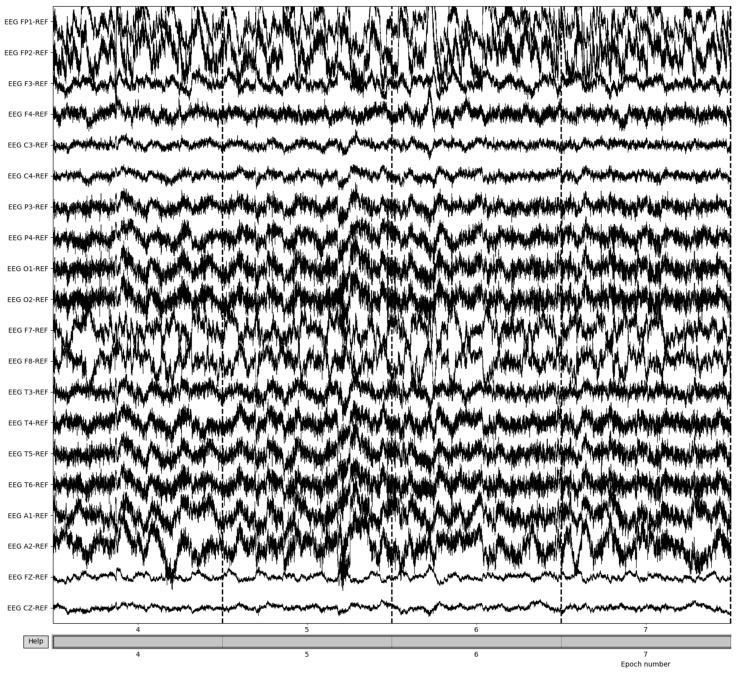
Post-bad epoch removal of EEG recordings.

**Figure 8:**
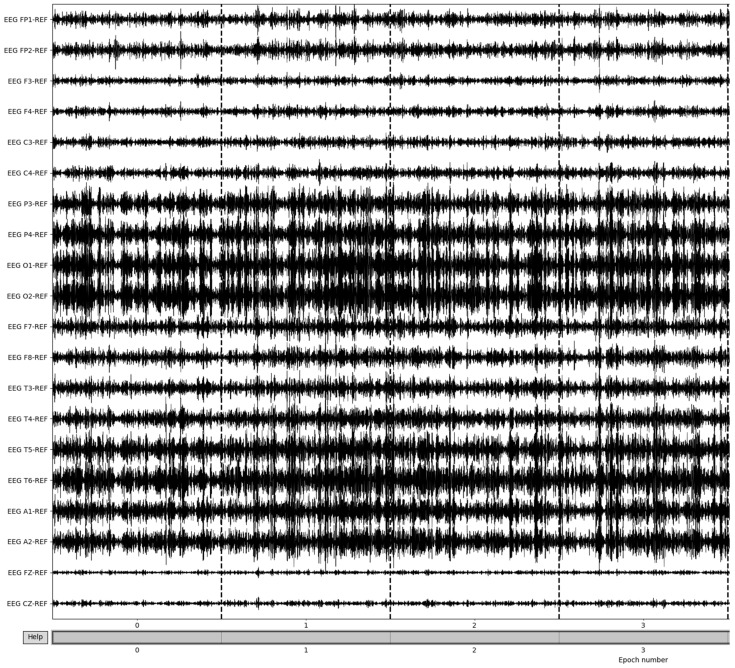
Beta frequency band post-bad epoch removal.

Bad epochs are those that have recordings that fluctuate wildly across the thresholds of the dataset. By utilizing the MNE-Python package’s drop_bad function, these epochs are removed, with the remaining epochs being used in further preprocessing. The number of epochs resulting from this removal varied from patient to patient based on the differences in the large fluctuations in the brain waves’ recording potentials. Figure 7 illustrates the result of bad epoch removal process. The process is completed by obtaining the frequency bands from the preprocessed signal, as shown in Figure 8.

**Figure 9:**
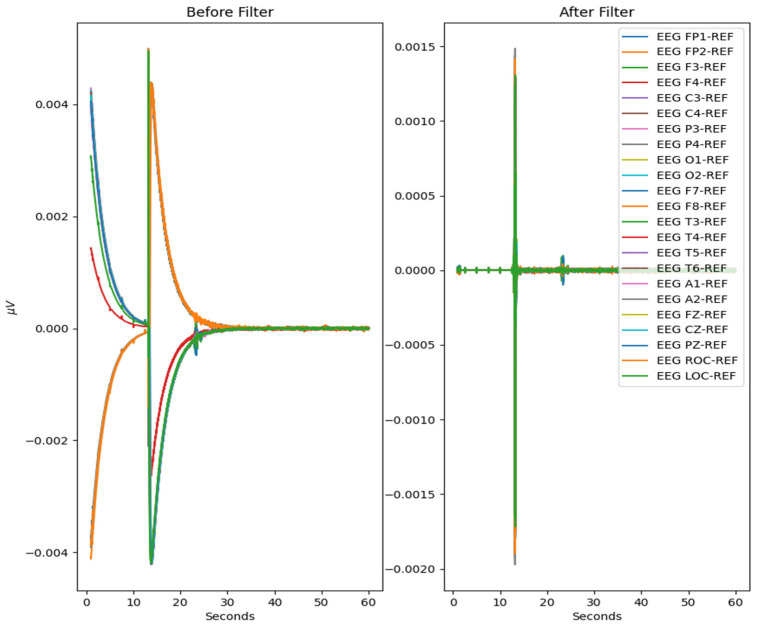
Difference in extraction of various frequency bands.

To obtain the frequency bands from the now preprocessed dataset, a bandpass filter is utilized. Using a high pass filter of 15 Hz and a low-pass filter of 35 Hz, the resulting beta frequency band can be obtained. To gather the other frequency bands, the low-pass and high-pass filters must match the ranges of the desired frequency bands; in the case of alpha frequency bands, a high pass filter of 8 Hz and a low pass filter of 15 Hz are utilized. The epochs of beta frequencies are a result of selecting only the clean epochs from the previous removal and extracting the frequency information (Figure 9). Though this method allows for the retrieval of the frequency band and epoching of the dataset, there are more methods to obtain the desired frequency bands that make up the patient brainwaves, which could be explored in further evaluations and yield more intricate details of the recordings.

Preprocessing is also undertaken to normalize the output data in certain instances during testing, reducing the range of values from the filtering methods. In the instance of testing the raw EEG signals, the data from the removal of the bad epochs are utilized. The reasoning behind this procedure is based on testing the ability of the deep learning architectures to extract features and detect trends in the raw data and make comparisons between more widely accepted preprocessing techniques and the more recent increased popularity of using of deep learning recurrent neural networks.

Each data file containing a desired neurodegenerative disorder undergoes either process depending on which preprocessing technique is to be evaluated. The extracted data points are saved into a single CSV file for storage and use for training and testing of the four deep learning models. The created CSV files contain the patient file info as an index, followed by the preprocessed/raw data and the patient age information that was extracted from the clinical text file either in a categorical or raw state. To form the categorical aspect of the prediction, the ages of the patients in the dataset are placed into specific age ranges.

### 2.4. Age Ranges

Since the patient information varies widely in the represented ages, the potential outputs and training data of the model should be simplified. Age ranges of the patients as well as the raw patient ages are utilized to evaluate both potential types of model outputs.

Initially, a total of 8 classes of patient ages are chosen, as seen in Table 1, and include patients of every age range, focusing primarily on the early brain developmental stages as well as later patient ages. Middle-aged patients within the range of 30–50 years old are initially less of a focus, as the range between the ages is much larger than those of the younger or older patients. Major brain development is already completed for middle-aged subjects and those in younger and older age ranges are most susceptible to neurodegenerative complications. However, to create a more balanced dataset for classification, the patients 30 years of age and above are utilized (Table 2). For example, subjects within the 30–45 years of age range represent 32% of the patients of the abnormal EEG corpus with a history of epilepsy or seizure (Table 1). While the dataset from the Temple University EEG Corpus does contain data from patients over 90+ years of age, the actual number of subjects represents a small percentage that would affect the balance of the training dataset. Training and testing are also performed on the raw ages of the patients, changing the problem from a classification problem to that of a regression analysis to predict the patients’ ages in years.

## 3. Deep Learning Models

Deep learning is a subset of machine learning that emulates multiple layers of processing to extract higher levels of features from data, emulating the way the human brain gains knowledge. Deep learning algorithms utilize gradient descent as well as backpropagation to self-adjust for increased precision in predictions. Deep learning models consist of multiple layers of interconnected nodes building upon each other to refine predictions through a powerful and intricate process, requiring a high amount of computing power and potentially requiring the use of GPUs.

It should be noted that in the related literature, convolutional neural networks (CNNs) are more frequently used alongside EEG data. A CNN model architecture is utilized as a baseline to compare its effectiveness against that of the proposed RNN-based approach in this work.

### 3.1. Recurrent Neural Networks

As an EEG recording develops over time, we utilize the capabilities of recurrent neural network (RNN) architectures to predict time series information. Each model is trained by leveraging an Nvidia RTX 2070 GPU to increase computational speed and the quick evaluation of model results. 

RNNs primarily focus on sequence-processing tasks and are commonly used for problems such as language translation or speech recognition. RNNs are distinguishable from feedforward networks and CNNs because they have a form of “memory” that utilizes prior information during training to influence the current input and output. RNNs also differ from the more traditional deep learning methods in that the output of the recurrent network depends on the prior elements in the sequence as opposed to assuming that the input and outputs are independent of each other.

**Figure 10:**
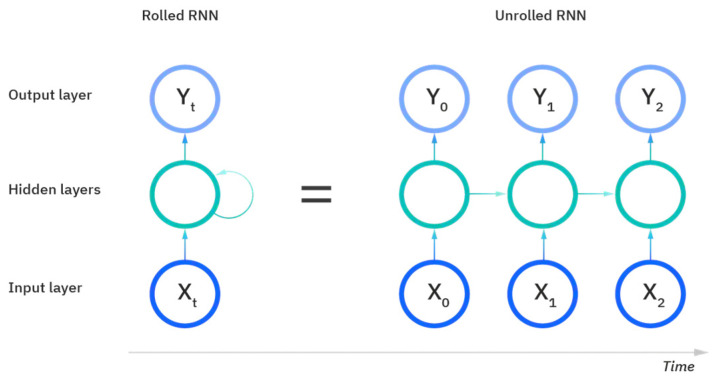
The rolled vs. unrolled visual of a Recurrent Neural Network (RNN) [24].

Figure 10 displays the setup of an RNN and its process of learning and prediction. The inputs to an RNN include the samples of data, the timesteps to evaluate and learn, and then the number of features evaluated over those timesteps to obtain the output. The rolled visual displays the entire RNN, with the unrolled version representing the entire input to the dataset. The hidden layers contain the information across the different timesteps that feed across the network. The recurrent unit of the RNN receives two inputs: the input at the current timestep and the hidden state of the previous timestep that is represented by the horizontal arrows. At the third timestep in this visual, the prior two timesteps are represented as hidden states used to predict the output in the sequence. The weights in RNNs are still adjusted similarly to feedforward networks; however, RNNs share the same layer weights across each node.

**Figure 11:**
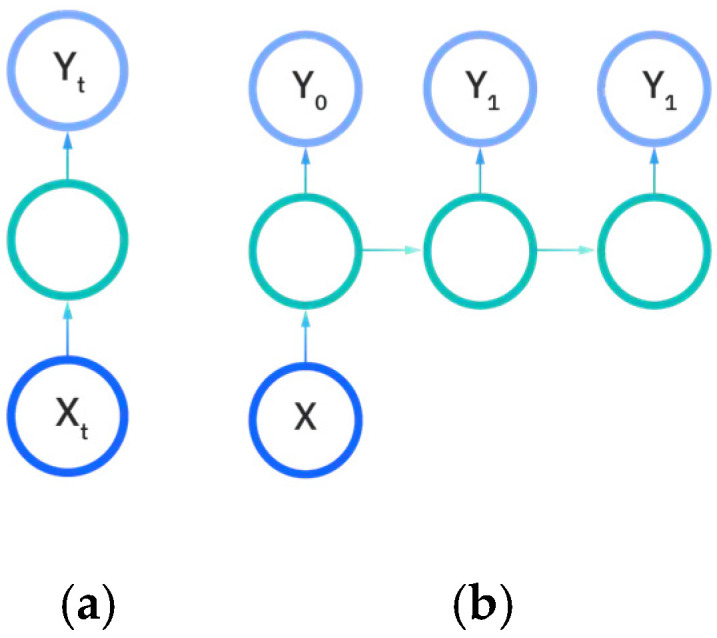

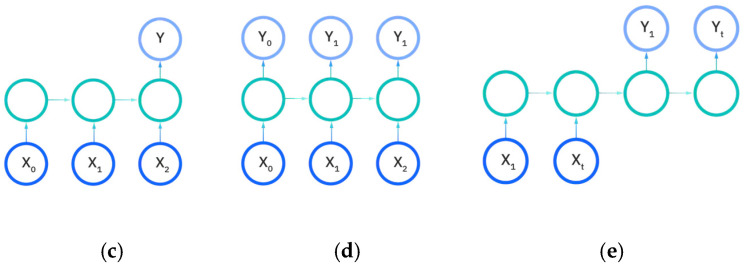
Input and output dimensions in RNN: (**a**) One-to-One; (**b**) One-to-Many; (**c**) Many-to-one; (**d**) Many-to-many; (**e**) Many-to-many (future prediction) [24].

There are five types of recurrent neural networks that denote the number of input and output dimensions as illustrated in Figure 11a–e. One-to-one and one-to-many RNNs (Figure 11a,b) take a single input feature and perform processes to predict either one output or multiple different feature outputs respectively. The same could be said about the many-to-one and the many-to many variants of the RNN (Figure 11c,d), as they utilize more than one input feature to either predict an output for one desired feature, or output to many features either in a one-to-one input to output ratio or in a sliding window. The recurrent neural network models developed in this work are either the many-to-many or many-to-one variations, as multiple EEG signals are used as input to either predict a vectorization of the categorical patient age or the raw patient age. We utilize the Figure 11e model for predictions.

#### 3.1.1. Long Short-Term Memory

The LSTM architecture, first proposed and developed by Hocreiter and Schmidhuber in 1997, addressed the time-consuming information storage over time intervals due to insufficient decaying error backflow [25]. The proposal of LSTM stems from other methods of recurrent learning or back-propagation tending to cause error signals “flowing backwards in time” to either “blow up” or vanish, meaning the temporal evolution of the error exponentially depends on the size of the weights. The LSTM could learn to bridge time intervals in excess of 1000 steps even in the case of noisy input sequences without the loss of time lag capabilities. The advantages of the LSTMs include their ability to bridge long time lags and handle noisy data. The two authors also state that the LSTM’s parameters have very little need for fine tuning such as a modification to a learning rate.

**Figure 12:**
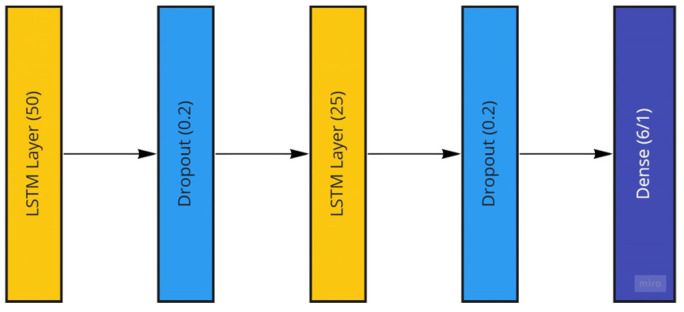
The Long Short-Term Memory Recurrent Neural Network Model utilized featuring two LSTM layers.

The LSTM model generated to be used with the Temple University dataset consists of two stacked LSTM layers followed by a dense layer for the model output. The two LSTM layers have sizes of 50 and 25, which are reached through systematic empirical experimentation. These two LSTM layers are followed by dropout layers to avoid possible overfitting and then a dense layer with a single node to account for the desired single number output of the patient age or a size of 6 to account for the six patient categorical ages located in the dataset. The LSTM model is trained using the ADAM optimizer with mean-squared-error loss and mean-absolute-error as well as root-mean-squared error accuracy measures for regression age prediction. More information on the performance measures is provided in the further sections. In the case of age classification, categorical measures for both loss and accuracy are utilized. The LSTM model is trained using both the raw EEG signals and the extracted frequency information from the preprocessing steps. The implemented LSTM model is conceptualized in Figure 12.

#### 3.1.2. Deep Bidirectional LSTM Model

The model we use is inspired by the work of Kaushik et al. [22]. The model in [22] is trained using the five different brainwave types, and it is found that the beta brainwaves along with the proposed model obtain the best results regarding accuracy for both the age and gender prediction overall. The model reports 97.52% accuracy for predicting gender with a maximum accuracy of 93.69% for predicting an individual’s age. As a direct comparison, our proposed approach and model recorded a 90% accuracy in predicting an individual’s brain age.

**Figure 13:**
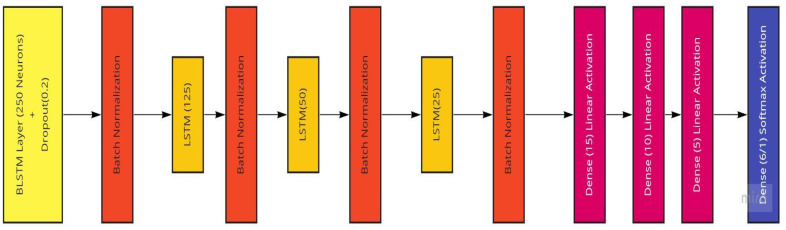
The BLSTM Model utilized featuring a BLSTM and three LSTM layers.

The model we use in this work (conceptualized in Figure 13) comprises an input layer bidirectional LSTM with 256 hidden neurons, followed by a 20% dropout layer and a batch normalization layer to ease overfitting to the training set, as well as three sets of LSTM and batch normalization layers with the LSTM layers having hidden node sizes of 128, 64, and 32, respectively. Following these layers are three hidden dense layers with sizes of 16, 8, and 4 to improve computational power. The output layer is a dense layer that varies in the number of neurons depending on the kind of analysis being performed. If there is a prediction of the raw patient ages as a regression and prediction, there would only be one node required for the expected one-feature output. In the case of the age ranges being categorized, the number of neurons in this final layer is equal to the number of unique age ranges, in this case six (Table 2). These model compositions underwent many variations as the result evaluation and testing continued. To improve the model’s performance, the use of dropout and batch normalization layers were employed to enable the model-training process to ease into a stable learning rate.

As batch normalization layers are used, the use of a much larger learning rate and batch size is feasible. Batch normalization is used on activations throughout the network as a form of scaling. This normalization prevents small changes to the parameters of a network from being amplified into larger and suboptimal changes in activations in the gradients [26]. Backpropagation through a layer is unaffected by the scale of its parameters due to batch normalization. When training with batch normalization active, a training example is seen in conjunction with other examples in the mini batch. This inhibits the training network from producing deterministic values for a given example [26]. There are many sources that either recommend batch normalization for use in recurrent neural networks to reduce training times and enable faster convergence [26] or deny their place in recurrent neural networks. Batch normalization layers are applied in this work to keep it uniform with related EEG age prediction research for purposes of comparison.

The learning rate for our model is 1 × 10^−5^, trained over 500 epochs with a batch size of 256 using 348 different EEG recordings. These sizes can produce a minimum loss much more efficiently during training. These parameters were chosen after the stages of testing were performed during the model-training evaluation. A higher learning rate would cause the deep learning model to learn too quickly and lose the necessary important information obtained from the chosen EEG channels as features, causing the model to become overfit to a local minimum of information. With a learning rate that is too low, the model would cease to learn any relevant EEG signal information, causing the results to be wildly different than was expected. We also experimented with different batch sizes and epoch numbers. Lower epoch numbers with higher batch sizes would cause the model to evaluate too many EEG signals at one time, causing a loss in its learning capabilities. With the higher learning rate and lower batch size that were ultimately chosen, the model can learn from the training dataset and be fit to a stable amount of information.

### 3.2. Convolutional Neural Networks

Convolutional Neural Networks (CNNs) are another type of deep learning network architecture with a primary use in classification tasks. CNNs are typically built with four types of layers: a convolutional layer, a pooling layer, an activation layer, and a fully connected layer for output [20]. CNNs are well-suited for end-to-end learning or learning from the raw data without feature selection and scale well with large datasets, allowing for the exploration of hierarchical structures in natural signals. The major disadvantage of CNNs is their tendency to output false predictions with high confidence. CNNs also may require a large number of training data and take much longer to train than simpler models [20].

The use of CNNs has been much more prevalent in the evaluation of EEG signal data as compared to RNNs. The Shallow Filter Bank Common Spatial Pattern Network (SFBCSPN) developed by Schirrmeister [20] is utilized as a benchmark, while the SFBCSPN [19] is used for EEG age prediction.

The preprocessing methods undertaken by Engemann differ from our approach presented in this work. The authors in [19] convert the EEG signals into a Brain-Imaging Data Structure (BIDS) data format, which enables the standardization of the neuroimaging data and interoperability. First, the data are bandpass-filtered between 0.1 and 49 Hz; then, they are split into 10-second epochs to coincide with the eyes-closed or eyes-open resting-state conditions. The amplitudes of the recorded EEG signals are automatically rejected. The data from the TUAB used in the evaluations in [19] consist of 21 common channels, and the dataset is resampled to 200 Hz for consistency. To maintain simplicity, the first recording from every patient is used. The data were post-resampled to evaluate the deep learning CNN. The model used in [19] and its resulting MAE value of 8 years of age are utilized as comparative measures for the deep learning recurrent neural network architectures employed in this paper. As a direct comparison, our proposed method achieves an MAE value of 7 years of age.

## 4. Results and Discussion

This section details the overall results of the models considered as well as the metrics used to evaluate them. A total of 388 EEG recordings were utilized from the TUAB Corpus with ages that fall in the age ranges from Table 1. A 70/20 train/test split was utilized for the model’s inputs and evaluation, with 10% of the training dataset being used as a validation set during training. Therefore, the training dataset consists of 279 EEG recordings. The testing dataset for the model’s evaluation consists of 78 EEG recordings. The validation set comprises 31 EEG recordings.

Each of the models is trained with a variable learning rate due to the model-checkpointing evaluation utilizing learning rate decay and early stopping. Learning rate decay is enabled to prevent a local minimum, which presents a plateau in the training process. The learning rate decay is enabled for every five epochs where there is no change in the validation loss. After the model begins to stagnate for five epochs, the learning rate is divided by half and training continues. The minimum loss of this learning rate reduction is set at 5 × 10^−7^. The batch size is also tuned to fully exploit the model’s capabilities and may differ between models due to the model’s architecture. The loss function of the models differs depending on the type of output. When dealing with data containing the raw patient ages from individuals as a regression problem, the mean squared error is utilized with the root mean squared error and the mean absolute error as accuracy measures. When categorical patient ages are utilized, the categorical cross-entropy is evaluated as a loss function, with the categorical accuracy measuring the accuracy. Further details on the metrics are provided in the following subsection.

When the patient age ranges are utilized, label encoding and categorization are employed to one-hot encode the patient ages into vectors. One-hot encoding removes the integer-encoded variables from the label-encoding process and adds a new binary variable for each unique integer value. In the case of the age ranges, for an age range of 41–45, a one-hot-encoded vector would be [0, 0, 1, 0, 0, 0], denoting the third age range category.

**Figure 14:**
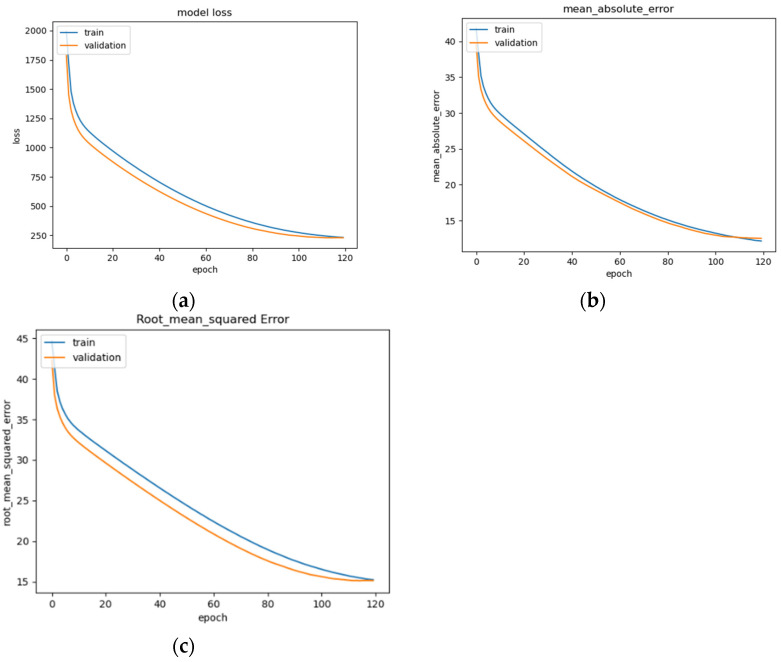
LSTM model performance: (**a**) Loss (MSE) of LSTM trained on EEG data with regression analysis; (**b**) MAE of LSTM trained on raw EEG data with regression analysis; (**c**) RMSE of LSTM trained on raw EEG data with regression analysis.

**Figure 15:**
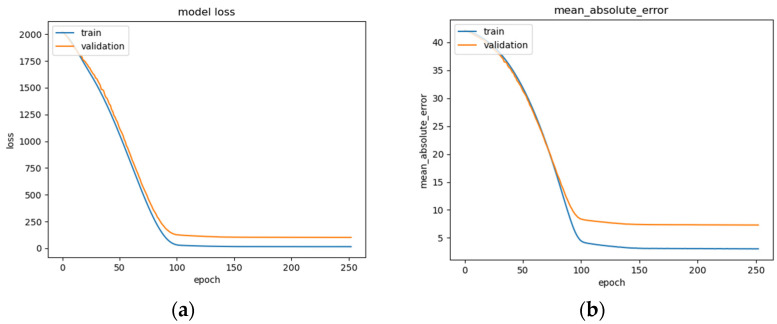

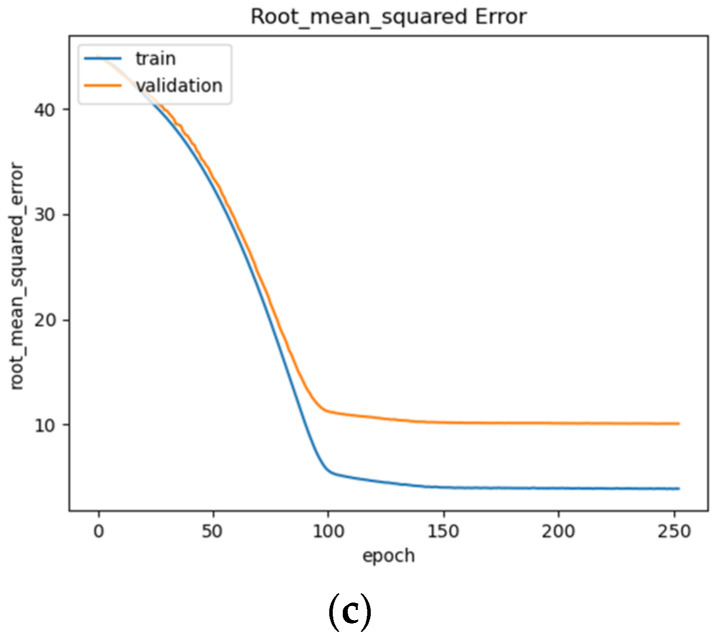
BLSTM model performance: (**a**) Loss (MSE) of BLSTM model trained on raw EEG data with regression analysis; (**b**) MAE of BLSTM model trained on raw EEG data with regression analysis; (**c**) RMSE of BLSTM trained on raw EEG data with regression analysis.

Many variations of the presented models have been tested in this work, with the best-performing models presented below in the following subsections. The graphs in Figure 13, Figure 14 and Figure 15 display the loss and accuracy measures as the training proceeded on the training and validation sets of EEG data. Table 2 shows the distributions of the average real age of the testing data versus the average mean error of the age predicted by each deep learning model in the cases of regression analysis. For age classification, the categorical accuracy percentage is utilized and shown in Table 3, with the precision, recall, and F1 model scores located in Table 4.

### 4.1. Evaluation Metrics

When performing regression analysis using the raw patient ages, the metrics utilized to evaluate the models are the mean squared error for the loss, as well as the mean absolute error, and the root mean square error for the accuracy measure. When evaluating the classification of patient age ranges, the categorical-cross entropy loss and categorical accuracy are used as metrics.

#### 4.1.1. Regression Metrics

Mean squared error (MSE) is the default loss function utilized for regression problems. The MSE denotes the mean overseen data of the squared differences between the true values and predicted values. With *y* as the actual value and $\hat{y}$ as the predicted value, the formula for calculating the MSE [27] is

(1) $$MSE=\frac{1}{m}\overset{n}{\underset{i=1}{\sum }}\;{\left(y-\hat{y}\right)}^{2}$$

The MSE is utilized when evaluating normally distributed data and essentially compresses all the training data and model predictions into a single value to determine how well a model can reproduce reality [20]. Models that predict a continuous variable, such as patient age, utilize the MSE as an ideal performance benchmark.

The mean absolute error (MAE) is an accuracy metric utilized for regression analysis. The MAE originated from a measure of average error. The MAE of a model displays the absolute value of the difference between the forecasted value and the actual value. The MAE of n samples of model errors ϵ is calculated with the formula [27]

(2) $$MAE=\frac{1}{m}\sum_{i=1}^{m}\;\left|y_{i}-\hat{y_{i}}\right|$$

Utilizing the MAE during model training gives the same weight to all errors, which differs from the root mean square error evaluation [28]. The MAE is less sensitive to extreme values than the MSE [29].

The root mean square error (RMSE) measures the average magnitude of the error, or how concentrated the data are around the line of best fit. The RMSE and MAE are often compared with respect to their overall effectiveness for evaluating model performance. Some sources differ in their analysis of MAE or RMSE being more suitable for measuring average model accuracy, as in [28]. The RMSE has been used as a standard statistical metric to measure model performance in meteorology or climate research studies [28]. Unlike the MAE, the RMSE penalizes large errors. The RMSE of *n* samples of model errors ϵ is calculated with the formula

(3) $$RMSE=\sqrt{\frac{1}{n}\overset{n}{\underset{i=1}{\sum }}\;{\left(S_{i}-O_{i}\right)}^{2}}$$

The use of the RMSE is very common and it is considered an excellent general purpose error metric for numerical predictions [30].

#### 4.1.2. Classification Metrics

Categorical-cross entropy is the most common loss function for the evaluation of multi-class classification problems. Categorical data refer to observations y that take values in a discrete sample space Ω formed by K distinct elements using K one-hot vectors [31]. The cross-entropy loss is equivalent to the negative log-likelihood of y∈ Ω under a categorical distribution with parameter π. Cross-entropy is displayed as the function

(4) $$l\left(\pi ;y\right)=-\sum_{k=1}^{K}\;y_{k}log\pi_{k}\Leftrightarrow \;p\left(y;\pi \right)=\prod_{k=1}^{K}\;\pi_{k}^{yk}.$$

Cross-entropy loss defines a coherent probabilistic model for discrete data over K classes [31].

The final employed evaluation metric is the confusion matrix that is used to define the ability for the age classification models to generalize on the testing data subset. A confusion matrix is commonly utilized to analyze the potential of a classifier.

A classifier is often also evaluated using three other metrics: precision, recall, and the F1-score. Recall is calculated by taking the proportion of the correctly identified positive inputs, also known as the TP rate, given by Equation (5):

(5) $$\mathrm{Recall}=\frac{TN}{FP+TN}$$

Precision is the correctly predicted positive cases by the classifier measured by Equation (6):

(6) $$\mathrm{Precis}=\frac{TN}{FN+TN}$$

The F1 score is the measure of the test’s accuracy using a weighted mean of precision and recall. The maximum F1 score is 1, with the worst score being 0. The F1 is defined using Equation (7):

(7) $$F1=\frac{2x\;Precis\;x\;Recall}{Precis+Recall}$$

The effectiveness of a classifier is determined by the values generated from the matrix as well as the subsequent recall, precision, and F1 scores [32].

Another form of classification accuracy analysis comes in the form of the receiver operating characteristic (ROC) curve. The ROC curve is a graph displaying the performance of a classification model at all classification thresholds, plotting the recall and false positive rates at different classification thresholds. In order to evaluate the ROC curve, the use of a sorting-based algorithm is utilized, which is known as the area under the ROC curve (AUC). The AUC provides the aggregate measure of performance across all possible classification thresholds. The AUC is the probability that the model ranks a random positive example more highly than a random negative example. The AUC is scale-invariant, measuring how well the predictions from the deep learning model are ranked rather than their absolute values. The AUC takes values from 0 to 1, where a value of 0 indicates a perfectly inaccurate test, with 1 representing a perfectly accurate test. A value of 0.5 suggests no discrimination, or no ability to make a classification of patient age ranges based on a test. The diagonal line presented in the ROC curve graphs display shows the threshold for the deep learning model’s discrimination ability, with ROC curves above the line showing a better ability to determine patients’ brain ages [33].

### 4.2. Experimental Results

The tested models can be organized into two distinct sections: those utilizing two basic layers of LSTM or GRU, and bidirectional layered models utilizing batch normalization. All the models are trained over 500 epochs, with a learning rate of 1 × 10^−5^, and a batch size of 256 to maximize the use of the GPU. These hyperparameters are chosen to form a basis for the deep learning models’ evaluations, ensuring that all the models can be compared under the same circumstances. These larger learning rates and batch sizes are utilized to make the most out of the batch normalization layers. Learning rate decay and early stopping are employed to stop the model from stagnating during training. Learning rate decay is activated after the deep learning model has begun to plateau in terms of learning the information given. After three epochs, if the model training accuracy or MAE have not shown positive performance, the deep learning model architecture will decrease the learning rate further down by half of the current rate. This continues until early stopping activates. Early stopping evaluates the models’ proficiency after five epochs, monitoring the validation loss of the model-training parameters. After five epochs, the model will cease its learning and the resulting deep learning model will be saved. While GRU and bi-directional GRU models have been researched, due to a lack of significant gains in accuracy, these models are not included in the discussion.

#### 4.2.1. Regression Experimental Results

Regression attempts to determine the strength of the relationship between one dependent variable and any number of independent variables. The independent variables in this case are the EEG recordings from the 23 recording devices. The dependent variable, therefore, is the patient’s chronological age obtained from the text files accompanied with the EDF recording files.

The LSTM model’s performance is illustrated in Figure 14a–c, showing convergence around epoch 120 before early stopping occurs (Figure 14a). The curve of the data suggests that the model fits well for the input data (Figure 14b). The RMSE of the model after training is 15.1 (Figure 14c). This is a basic version of an LSTM model that can be commonly seen across deep learning resources, and as such, is implemented and trained for the evaluation of the other models as a benchmark.

The BLSTM’s performance is shown in Figure 15a–c, illustrating a steady decrease in training loss before beginning to plateau around epoch 100 (Figure 15a), after which early stopping occurs at epoch 250. The BLSTM model achieves an MAE of 7.3 years of age (Figure 15b) and an RMSE of 10.07 (Figure 15c). This MAE is an improvement over the MAE of 8 years being used as a direct comparison and utilizing the same dataset. This difference could be due in part to differences in the organization of the EEG recordings and preprocessing techniques employed such as high and low pass filter values, epoch-splitting ranges, and changes in the frequency band ranges.

#### 4.2.2. Age Classification Experimental Results

Classification analysis, unlike regression analysis, attempts to predict a class label for an evaluated set of data. The patient ages were grouped as shown in Table 2. Though this does reduce the number of samples of the dataset, it leads to a more evenly distributed dataset, which is imperative for training a model for classification. However, to form an evenly distributed dataset, the ranges that occur less often than the highest occurring age range are oversampled for a balanced dataset. The patients’ age ranges are transformed into one-hot vectors for classification output. These models differ in architecture from those utilized in the regression analysis in that the output dense layer has six nodes, one for each possible class, instead of the one for a single output.

**Figure 16:**
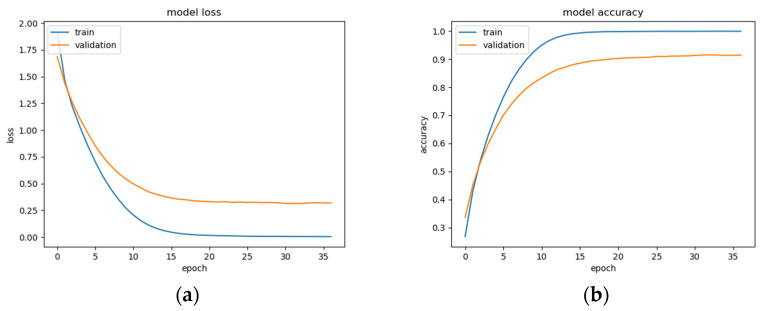
BLSTM model performance: (**a**) Categorical cross-entropy loss of the utilized BLSTM model on the raw EEG data; (**b**) categorical accuracy of the utilized BLSTM model on the raw EEG data.

**Figure 17:**
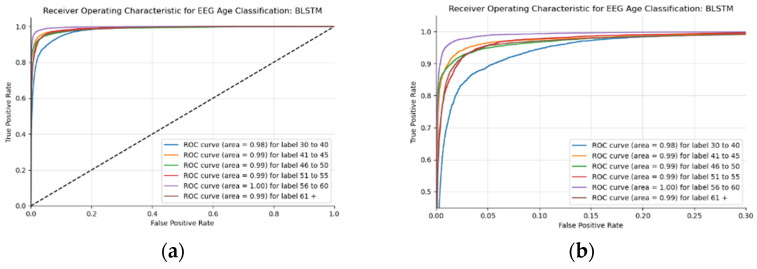
ROC of the utilized BLSTM model on the raw EEG data: (**a**) full ROC figure; (**b**) zoomed in portion to illustrate the initial characteristics of the experiment.

**Figure 18:**
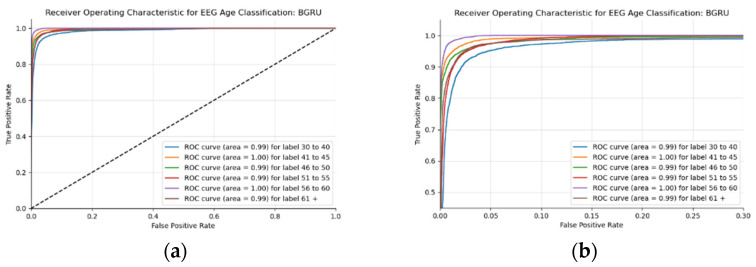
ROC of the utilized BGRU model on the raw EEG data: (**a**) full ROC figure; (**b**) zoomed in portion to illustrate the initial characteristics of the experiment.

This BLSTM model employed in this experiment records an accuracy of 91% validation accuracy over 35 epochs of training, achieving F1 and precision scores of 0.9, using the same hyperparameters from other tests. Though the graphs in Figure 16 show that overfitting to the training dataset does occur, there is no drastic increase in the training loss and subsequent accuracy measures. Figure 16a presents the model loss and Figure 16b illustrates the model accuracy. The possible explanations for the displayed overfitting include that the regularization and generalization techniques, dropout, and batch normalization utilized to counteract overfitting were not employed during the validation set’s testing by default. Figure 17a,b display the ROC curves of the employed BLSTM model with Figure 17b showing magnified details in the range of a 0–0.3 false positive rate. These figures display that the BLSTM model is excellent at discriminating between the six given age ranges, with variable AUC scores in the upper 90%. The employed BLSTM model can discriminate between the upper age range of 61+, with a much lower ability to classify the 30–40 age range. The BGRU’s ROC curves displayed in Figure 18a,b showcase the slightly higher discriminating ability of the employed BGRU model, which has a better ability to classify the lower 30–40 age range.

**Figure 19:**
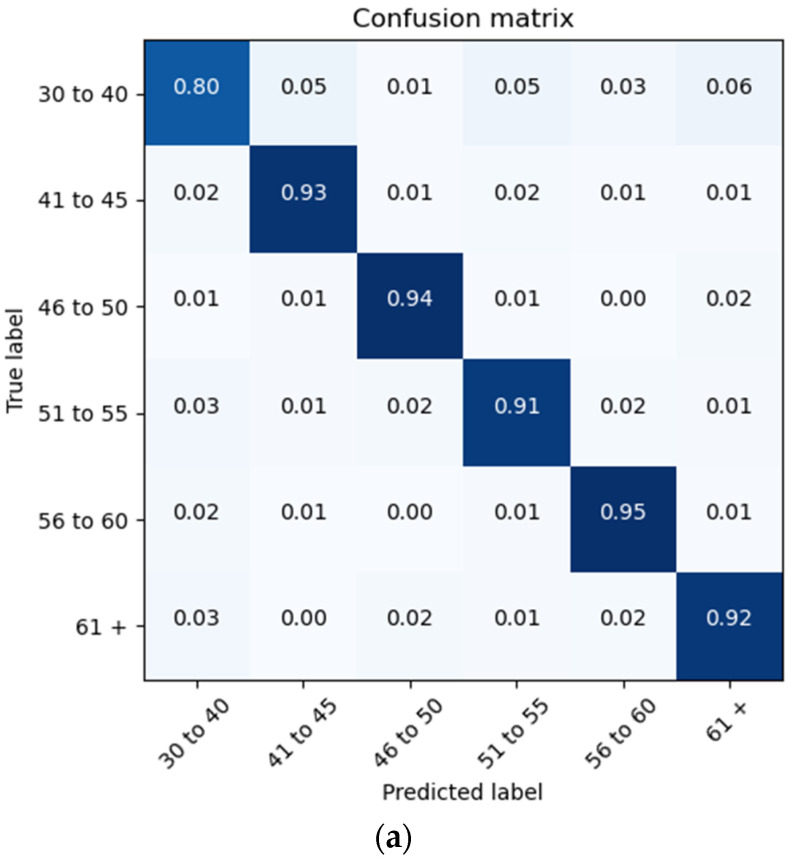

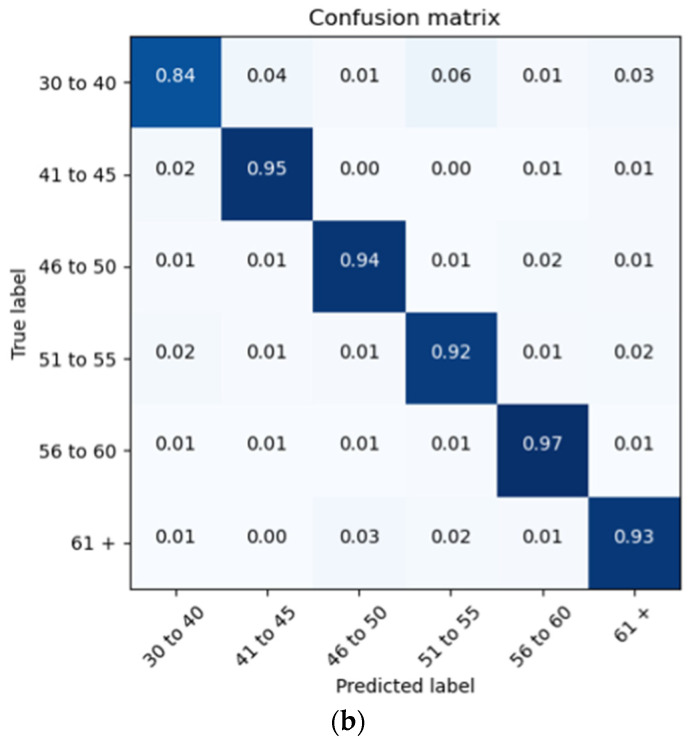
Confusion Matrices Generated from: (**a**) BLSTM raw data testing; (**b**) BGRU raw data testing.

The proposed architecture achieves a patient brain age classification overall accuracy of 90% (minimum of 80% and maximum of 95%) on the test data subset (shown in Figure 19a). The BGRU results are shown in Figure 17b, demonstrating a slightly better accuracy of 93% and exhibiting F1 and precision scores of 0.93 and 0.92, respectively (also shown in Figure 19b).

### 4.3. Discussion

There are few research publications on the prediction or classification of age using RNNs with EEG data. The aim of this work is to evaluate different RNN architectures with respect to their power in evaluating raw EEG data for predicting the ages of patients with seizure history. Obtaining the best deep learning model for this problem is an impossible task, as deep learning models can always be improved by the constant adjustment and re-evaluation of parameters. The number of possible implementations of model architectures is also large enough that finding the perfect working model for a problem cannot be achieved. Models can be improved by the addition or removal of layers and different activation functions within the layers, as well as by utilizing regularization techniques such as batch normalization [34] or dropout. The deep learning techniques employed in this research are based on many evaluations and modifications to the architectures, as well as an examination of the related literature.

These models can be compared to the SFBCSPN model employed by Engemann [19]. The models employed here could reach and even improve upon the same MAE value of the SFBCSPN model. The metrics achieved from the raw data overtake that of the SFBCSPN model, displaying the power of the LSTM to extract necessary features from the raw data themselves. The explanation for the higher metrics evaluated for the other preprocessing steps could involve the differences in the preprocessing methods employed. Engemann et al. utilizes a different frequency than that which is utilized in this work: 200 Hz versus 250 Hz. Engemann et al. [19] also utilizes 21 EEG channels commonly seen in the 10/20 system, while this work utilizes the extra T2 and T3 nodes, which could have caused an error in regularization. Other explanations could involve the data-preprocessing methods themselves, as Engemann et al. opted to transform the data into a brain-imaging data format (BIDS) for use in a CNN architecture instead of utilizing the raw data.

The RNN architectures employed in this research show some degree of the power of the LSTM and BLSTM architectures and their ability to extract important features from raw data, seen from the results in Table 3 and Table 4. The tested GRU models demonstrate a similar output to that of the LSTM variants with the added benefit of faster convergence. The presented models are trained with the true chronological patient ages as a training example for the patients’ brain ages. When evaluating the performance of the models on the datasets, the MAE should be higher than the patient age, as there is an MAE of 7 years.

The LSTM/GRU architectures can determine the significant information from the raw EEG recordings themselves without the need for any further signal-preprocessing techniques to be utilized. These other techniques include discrete wavelet transform (DWT) and fast Fourier transform (FFT) that are most commonly seen when attempting to extract information from signal-based data.

## 5. Conclusions

Electroencephalography is an electrophysiological monitoring method that is used to record the electrical activity of a patient’s brain. EEGs are primarily used to monitor the brain activity of those who have suffered from brain-related disorders—ranging from epilepsy to brain lesions—that are the result of a stroke. With an increase in the average lifespan around the globe, more patients are becoming affected by neurodegenerative diseases such as dementia and Alzheimer’s. One in ten individuals over 65 years old are diagnosed with Alzheimer’s, with the rate only increasing with age.

The study of how the brain changes in response to aging has been underway for decades. As people age, their brains shrink, and this affects all levels of biology from their molecules down to their morphology. This brain shrinkage often leads to an increase in blood pressure and with it an increase in the possibility of a stroke. The most common cognitive change associated with an aging brain is that which manifests in a person’s memory. Young patients diagnosed with epilepsy have been found to develop Alzheimer’s at a rate six times higher than patients without epilepsy.

BrainAGE is the first widely used method for predicting and evaluating a person’s chronological age versus their brain age. It utilizes structural MRI data to quantify the acceleration or deceleration of a patient’s brain. EEG-recording devices are less costly than MRI devices and are less complex. EEG recordings are available in both non-invasive and invasive forms. These EEG recordings are placed on the skull, often using a 10/20 system of EEG placement based on the distance between electrodes.

EEG recordings measure brain waves, which are rhythmic patterns in the central nervous system. Brain waves can be measured in both space and time, as they exhibit power and frequency. Various regions of the brain do not emit the same brain wave frequency simultaneously; brain wave patterns are unique to each individual patient.

The changes in the cerebral activity that come from an aging brain can be utilized to study the brain age versus the chronological age of a patient. This research focuses on recurrent neural networks and long short-term memories specifically to both categorize and predict a seizure patient’s brain age. RNNs are a research focus due to their noted performance regarding time-related information. Interestingly, there are very few related studies pertaining to the use of RNNs for brain age prediction. A vast majority of researchers are utilizing more typical machine learning approaches or implementing CNN architectures and finding success. The data from the Temple University EEG Corpus constitute a very comprehensive database of EEG recordings spanning a large variety of ages throughout a long period of time.

Six models have been examined, with the notable ones reported in previous sections. The comparison to the CNN model by Engemann et al. [19] with the Abnormal EEG corpus revealed that the CNN reached an MAE value of 8 years of age. The BLSTM and BGRU models examined in our work perform slightly better than the benchmark CNN model in terms of their regression prediction accuracy, reaching an MAE value of 7+ years of age. In terms of the categorical analysis, the proposed models and dataset utilized outperform Kaushik et al.’s 93.69% accuracy for age prediction by a little over 5%, for an accuracy of 99%. This may be due to the use of DWT employed in Kaushik et al.’s [22] experimentation. The use of DWT, which split the data into frequency bands using a formulaic breakdown of different ranges, could have caused some overlap between the recording frequency between each band. Kaushik et al. also utilized a different data source, choosing to gather the EEG recordings personally, utilizing different recording lengths and preprocessing techniques.

While finding a perfect model for a particular problem is not possible as the range of possible implementations is so vast, the advantages of the LSTM and GRU architectures cannot be ignored, as they are able to perform well enough in terms of the raw patient EEG data in the regression analysis, reaching MAE values close to and even improved from the benchmark, as well as performing quite well on this particular dataset regarding age classification. The employed architectures perform excellently with respect to the raw patient EEG data, with few other signal preprocessing methods employed. Many other researchers employ the use of signal-preprocessing techniques such as discrete wavelet transform or fast Fourier transform to extract any one of the five EEG frequency bands mentioned above. However, our methodology shows that these signal-preprocessing techniques are unnecessary, as the raw patient data with beta brain waves extracted by a simple bandpass filter suffice, leading to the results presented.

All the data utilized in this research originate from patients that have a history of seizures, either recently in life or those that have been seizure-free for any number of years. Many related works have obtained a personal database that had been personally gathered themselves or utilized a publicly available database of less varied recording ages and recording years. This process was not undertaken in this research due to a desire for the accessibility of anonymized patient information. While there are other publicly available EEG databases, such as the Boston Children’s Hospital database, they often do not contain the patients’ age information, are focused on a subset of younger demographic patients, or contain a very small subset of patient signals that would restrict model efficiency. The exploration of further preprocessing methods may be a way to further the accuracy of the employed models. While preliminary steps through FFT and DWT have been explored, the underwhelming results concerned the work on the raw EEG data and the power of the LSTM and GRU model architectures. Kaushik et al. [22] reports that beta brain waves are the best for age classification in their research utilizing 60 patient brain waves split into six age categories ranging from 6 years of age to 55 years of age. As such, an evaluation of other frequency bands or a combination of those bands could lead to better age classification or age prediction results for patients with seizure history. The use and further exploration of the effect of signal extraction or signal-preprocessing techniques remains to be undertaken With this, however, the employed deep learning architectures in combination with the EEG brain signal-processing methods undertaken allowed for an outcome of desirable results when attempting to predict or classify a patient’s age based on their brain signals. There may not be a need for more elaborate signal processing than is typically anticipated, as a simple band pass filter is sufficient for obtaining a desired frequency band, so long as the results obtained are similar or better than its contemporaries.

## Figures and Tables

**Table 1:** Initial patient age ranges.

Age Range	Approximate Percentage of Dataset
6–10	2%
12–15	0%
16–23	12%
24–29	11%
30–45	32%
46–55	24%
56–60	7%
60+	13%

**Table 2:** Revised patient age ranges.

Age Range	Approximate Percentage of Dataset
30–40	26%
41–45	17%
46–50	14%
51–55	18%
56–60	9%
61+	17%

**Table 3:** Results of each model from regression analysis.

	MAE	RMSE
LSTM	12.5	15.1
GRU	12.2	14.8
BLSTM	7.3	10.07
BGRU	6.5	9.1

**Table 4:** Results of each model with respect to categorical accuracy.

	Train	Valid	Test	Recall	Precis	F1 Score
BLSTM	99%	91%	90%	0.9	0.9	0.9
BGRU	99%	93%	93%	0.93	0.92	0.93

## Data Availability

The dataset can be found in the Temple University EEG repository.
